# Common variable immunodeficiency disorders: What generalists should know

**DOI:** 10.7196/AJTCCM.2021.v27i3.228

**Published:** 2021-10-04

**Authors:** A C Jeevarathnum, A van Niekerk, J Kriel, R J Green

**Affiliations:** 1 Department of Paediatric Pulmonology, Faculty of Health Sciences, University of Pretoria, South Africa; 2 Sandton Allergy and Immunology Centre, Johannesburg, South Africa; 3 Department of Paediatrics and Child Health, Faculty of Health Sciences, University of Pretoria and Steve Biko Academic Hospital, Pretoria, South Africa

**Keywords:** immune deficiency, common variable, predominant antibody deficiency

## Abstract

Primary immune deficiency disorders (PIDDs) are common and underdiagnosed. Predominant antibody deficiencies (PADs) are the most common type of immune deficiency and comprise 55% of the immune deficiencies diagnosed.^[1]^ Although immunoglobulin A (IgA) deficiency remains the most common type of PID, common variable immunodeficiency disorders remain the most common symptomatic PID for which medical therapy is sought.

## Background

Hypogammaglobulinaemia is the hallmark of common variable immunodeficiency disorders (CVID), where the loss of B-cell function is the causative immune defect observed in all patients. CVID is not a single disease but rather a heterogeneous group of primary antibody failure syndromes, defined by hypogammaglobulinaemia, increased susceptibility to recurrent and chronic infections, and impaired functional antibody responses.^[1]^ Biochemically, this common phenotype manifests with reduced levels of immunoglobulin G (IgG) and eitherlow IgA and/or low IgM, together with an impaired antibody response to vaccines.^[2–4]^

Individuals with CVID are susceptible to recurrent infections with mainly encapsulated extracellular organisms and are more prone to autoimmune diseases, lymphoproliferative disorders, and malignancies. Usually, the diagnosis is made between the ages of 20 and 40 years; however, up to 20% of patients may present before the age of 20 years. The onset of clinical manifestations and laboratory findings do not always coincide and can occur from early childhood to caducity.^[3]^

In this review article, we review the latest literature on CVID, particularly the epidemiology, pathophysiology, clinical features, differential diagnosis, diagnostic criteria, and association with autoimmune and lymphoproliferative disorders. The difference in presentation between adults and children is explored and basic treatment strategies are discussed.

The purpose of this overview is to increase awareness of this heterogeneous group of disorders, facilitating early diagnosis and appropriate management to mitigate the devastating sequelae of untreated CVID.

## Epidemiology

CVID was first described in 1954 in a 39-year-old woman with a gammaglobulinaemia and numerous pulmonary and gastrointestinal complications.^[2]^ CVID has no gender predilection and occurs at an incidence of 1:10 000 to 1:50 000 in a population,^[5–7]^ and has a bimodal age distribution with diagnostic peaks in childhood and early adult life.^[8]^ One-fifth of patients are diagnosed before the age of 20 years.^[8]^ It certainly is the most common primary immune deficiency disorder (PIDD) detected in the adult population.^[9]^ There is usually a delay of 4 - 6 years before the diagnosis is confirmed because of the heterogeneous array of clinical presentations.^[5,9,10]^

## Aetiology

Most cases of CVID are sporadic. There is, however, a genetic basis in 10 - 20% of cases.^[5]^ Patients who develop CVID because of a genetic predisposition usually inherit the disorder in an autosomal dominant fashion.^[5,9]^ Research has shown several HLA associations.^[5,9]^ It has been estimated that 15% of patients with CVID will have a first-degree relative with IgA deficiency or CVID. In addition, there might be a genetic association between CVID and IgA deficiency, with both disorders reflecting variable expression of a common genetic defect.^[5]^

## Pathophysiology

CVID involves immune dysfunction of T and B cells in addition to dendritic cells. The basic pathophysiological mechanism underlying the disorder is the failure of B cells to differentiate into plasma cells, resulting in reduced production of Igs. The absolute number of B cells might be normal, but the cells are dysfunctional. Because of the reduction in production of Igs, the adaptive immune system is compromised, and the patients are prone to developing recurrent infections. The more intricate details of the pathophysiology of the disorder are beyond the scope of this article.

## Clinical manifestations

Most patients with CVID will have at least one of the varied clinical presentations: infection and lymphoproliferation or autoimmunity, but as noted before, the phenotype is varied and may range from bacterial infections only to severe illness such as a combined immunodeficiency.

Patients may also present only with autoimmune disease, granulomatous disorders, or enteropathy with no evidence of recurrent bacterial infections.

The most common clinical manifestations include six categories, each of which will be discussed separately. A summary of clinical manifestations and complications is provided in Table 1.^[4,5,9,11,12]^

**Table 1:** Summary of clinical manifestations and complications of CVID

**Infections**	**Otitis media**
**(Predominantly respiratory and gastrointestinal)**	Sinusitis Pneumonia Recurrent pneumonia Gastritis Diarrhoea
**Chronic pulmonary conditions**	Bronchiectasis Bronchiolitis obliterans Asthma Granulomatous infiltrations Lymphocytic interstitial pneumonitis
**Diffuse granulomatous disease**	Noncaseating granulomatous of the lung, liver, spleen, bone marrow, lymph nodes, skin, kidney, eyes and brain.
**Gastrointestinal disease**	Infective diarrhoea Chronic diarrhoea Malabsorption Inflammatory bowel disease Autoimmune hepatitis Primary biliary cirrhosis
**Liver disease**	Nodular regenerative hyperplasia Autoimmune hepatitis
**Autoimmune**	Autoimmune haemolytic anaemia Immune thrombocytopaenia Seronegative Rheumatoid arthritis Pernicious anaemia, alopecia, vitiligo Sjogrens syndrome Uveitis, vasculitis, Sicca syndrome, SLE
**Malignancy**	Lymphoma Gastric carcinoma

CVID = common variable immune deficiency

SLE = systemic lupus erythematosus

It should be noted that not all patients with CVID will develop complications owing to immune dysregulation. Some patients develop infection only phenotype (33 - 80%) and may also develop infection-related structural complications such as bronchiectasis but not autoimmunity, interstitial lung disease, granulomatous disease, liver and gastrointestinal inflammatory disease, and lymphoid hyperplasia or cancer.^[2,3,13]^

### Recurrent and unusual infections

Most patients with CVID have a history of acute or chronic sino-pulmonary infections, particularly sinusitis, otitis media and pneumonia.^[14–16]^ This is usually caused by encapsulated bacteria,^[14–16]^ and up to 75% of patients usually have an episode of community-acquired pneumonia prior to diagnosis. It is common to find patients who present with recurrent episodes of pneumonia.^[17,18]^ The most common pathogens include *Streptococcus pneumoniae*, *Haemophilus influenzae*, *Moraxella catarrhalis*, *Staphylococcus aureus* and *Pseudomonas aeroginosa*. *Mycoplasma* is also an important intracellular pathogen in patients with atypical respiratory tract infections. CVID is unlike PIDs that are prone to opportunistic infections with fungal or viral pathogens. 

Patients with CVID (10 - 40%) are prone to gastrointestinal infections with pathogens such as *Giardia lamblia*, *Campylobacter jejuni* and *Salmonella* spp., and *Giardia* is the most common pathogen.^[19]^ *Helicobacter pylori* is an important pathogen in CVID and causes chronic active gastritis.^[20]^

Unusual urinary and cervical infections owing to *Ureaplasma* urealyticum as well as meningoencephalitis or dermatomyositis-like syndrome caused by enteroviruses have been described in undiagnosed and untreated patients.^[21,22]^

### Chronic pulmonary conditions

It is estimated that between 37.5% and 73% of patients with CVID have bronchiectasis and it has been shown that patients with low levels of IgM are at increased risk of developing recurrent pneumonia and bronchiectasis.^[23,24]^

Airway inflammation is commonly seen and may progress to obstructive or restrictive disease given time. Asthma has been observed in 9 - 15% of patents with CVID. The actual aetiology of asthma in patients with CVID is largely unknown.

Mediastinal lymphadenopathy is common in CVID. Nonnecrotising granulomas have been described in 8 - 22% of patients with CVID; these non-necrotising granulomas differ from those from sarcoidosis as they are not located peri-lymphatically. Sarcoidosis is usually associated with hyper-gammaglobulinaemia.^[25–27]^ Lymphocytic interstitial pneumonitis has also been reported.

### Gastrointestinal disease

There is a high incidence of inflammatory and infectious gastrointestinal disorders in patients with CVID.^[28]^ Mild watery diarrhoea is common and occurs periodically. More severe clinical disease with malabsorption and weight loss are well described and occur in 10% of cases. Gastrointestinal pathology may include nodular lymphoid hyperplasia and autoimmune hepatitis progressing to portal hypertension. 

A challenging form of chronic small bowel inflammation and CVID-associated autoimmune enteropathy (AIE) occurs in up to 12% of patients presenting with unexplained persistent diarrhoea, malabsorption of minerals and fat-soluble vitamins, steatorrhoea, and weight loss. These patients often have vitamin A deficiency with added suppression of Ig production. Mild hepatomegaly and an elevated level of alkaline phosphatase is also seen in CVID.^[29–31]^

### Granulomatous or polyclonal lymphocytic infiltrative complications

Some patients (10 - 25%) with CVID develop granulomatous or lymphoproliferative diseases. The mean age of diagnosis is 20 - 40 years. Granulomatous lesions can affect any region of the body, but most commonly affect the lungs. Patients present with chronic shortness of breath and cough, which is not related to asthma.^[8]^

### Autoimmunity

It has been suggested that up to 30% of patients with CVID will experience an autoimmune process. Autoimmune disease was the sole clinical finding at the time of diagnosing CVID in 2.3% of patients.^[29]^ Common autoimmune entities seen in CVID patients include autoimmune cytopaenias such as immune thrombocytopaenic purpura (ITP), autoimmune haemolytic anaemia (AIHA), combination of ITP and AIHA (Evan’s syndrome), seronegative rheumatoid arthritis (RA), pernicious anaemia, inflammatory bowel disease, Sjögren’s syndrome, uveitis, vasculitis, thyroiditis, alopecia, vitiligo, primary biliary cirrhosis, and sicca syndrome or systemic lupus erythematosus.^[5,29]^

Common autoimmune diseases such as type I diabetes mellitus, seropositive RA, psoriasis, celiac and hypothyroidism are not increased in patients with CVID.

Not only is the underlying pathogenesis of CVID poorly understood, but the management of concurrent autoimmunity often becomes a challenging task. It is very important to emphasise that autoimmunity may be the presenting feature of CVID without any evidence of recurrent infections characteristic of a PIDD.^[32]^

There seems to be a higher prevalence of autoimmune processes in patients with a paucity of switched memory B cells (CD27+, IgM–IgD–). Another characteristic feature predictive of an autoimmune process in patients with CVID is the presence of granulomatous infiltrations in the lungs, nodes or other organs.

CVID patients with autoimmune diseases are treated using higher doses of immunoglobulin replacement therapy (IRT), and when immune suppressive drugs are administered, it is advised that lower doses and shorter courses are given to prevent the development of opportunistic infections considering the underlying PIDD.

### Malignancy

The lifetime risk of all types of malignancies in adults with CVID is around 6 - 9%.^[33]^ CVID patients are at tenfold increased risk of developing solid or haematological malignancies.^[33]^ Patients with lymphoma typically have a childhood onset and those with gastric tumours present later in adulthood, particularly in the fourth decade of life. The most common type of malignancy reported in patients with CVID is non-Hodgkin’s lymphoma.^[34]^

## Making a diagnosis

There is no single test or biochemical marker available to diagnose CVID. The diagnosis is made by a constellation of clinical features and several laboratory investigations. It is the combined result of these tests from which a diagnosis can be made. There are more common genetic disorders that manifest with CVID. Testing for this is possible but expensive. However, diagnosis is not dependent on genetic testing only.

The diagnosis of CVID rests on exclusion of other causes of hypogammaglobulinaemia (Table 2).

**Table 2:** Secondary causes of hypogammaglobulinaemia^[4,13]^

**Drug-induced**	**Antimalarial agents**
	Captopril Phenytoin, carbamazepime Anti-CD20 mAbs: Rituximab Glucocorticoids Sulfasalazine
**Single gene and other defects**	Ataxia telangiectasia AR forms of SCID and other combined immunodeficiency Hyper IgM syndromes Transcobalamin II deficiency and hypogammaglobulinaemia X-linked agammaglobulinemia X-linked Lymphoproliferative disorder (EBV-associated) X-linked SCID
**Chromosomal abnormalities**	Chromosome 18q-syndrome Monosomy 22 Trisomy 8 Trisomy 21
**Infectious diseases**	HIV EBV Congenital infections with CMV/rubella/Toxoplasma
**Malignancies**	Chronic lymphocytic leukaemia Non-Hodgkin lymphoma Monoclonal gammopathy
**Other systemic disorders causing excessive loss of immunoglobulins**	Nephrotic syndrome Severe burns Lymphangiectasia Protein-losing enteropathy

mAbs = monoclonal antibodies

AR = autosomal recessive

SCID = severe combined immune deficiency

EBV = Epstein-Barr virus

CMV = cytomegalovirus

Demonstration of hypogammaglobulinaemia and defective specific antibody responses or levels is imperative. It is difficult to make this diagnosis in children younger than 4 years of age as the hypogammaglobulinaemia in this age group is most likely because of a transient hypogammaglobulinaemia of infancy (THI). However, patients with THI can develop CVID, but the distinction between the two is difficult.

The American Academy of Asthma, Allergy, and Immunology in collaboration with the European Academy of Asthma, Allergy and Immunology and World Allergy Organization published a consensus document on the diagnosis of CVID in 2015.^[4,35]^ According to the document, a diagnosis of CVID can be made if the following criteria are met:

- Age >2 years
- At least one clinical manifestation (infection, autoimmunity, lymphoproliferation)
- Demonstration of hypogammaglobulinaemia on two occasions at least 3 weeks apart as below:
  - Marked decrease of IgG levels at least 2 standard deviations below mean for age and either;
  - Low IgA and/or IgM
- Low specific antibodies, absent isohaemaglutinins and/or failure to exert an adequate vaccine response, or significant waning of vaccine levels
- Exclusion of defined causes for hypogammaglobulinaemia according to a list of differential diagnoses (Table 2).

## Management

The most important part of managing these patients is to make a diagnosis. The astute physician will be confronted with several clinical syndromes and recurrent infections and making the diagnosis will require a high index of suspicion.

Once the diagnosis has been made, the physician can explore IRT, which is the mainstay of management of patients with CVID.

The aim of IRT is clinical improvement and resolution of recurrent infections, either by subcutaneous or intravenous immunoglobulin replacement routes. One usually needs to maintain the trough IgG level at a level >400 - 500 mg/dL and, to achieve this, doses of 400 - 600 mg/kg every 3 - 4 weeks are required, with higher doses needed for established complications such as bronchiectasis with a trough level of no less than 700 - 800 mg/dL.^[4]^

Prophylactic antibiotics are often prescribed for patients with CVID. The benefit of this strategy is still under review and the jury is out on the long-term sequelae. Options of prophylactic antibiotics include trimethoprim-sulfamethoxazole or macrolides and there are various regimens described.

The prognosis of patients with CVID depends on several factors including the frequency of infections, structural lung damage and development of autoimmunity and malignancy.

## Conclusion

CVID is the most common symptomatic PIDD. More so, it is the most common PIDD presenting in adulthood. Symptoms are usually heterogeneous and nonspecific. Patients present with recurrent infections caused mostly by encapsulated extracellular bacteria. Viral and fungal opportunistic infections do not often occur in patients with CVID. Diagnosis of CVID is by exclusion and molecular testing is not considered necessary for diagnosis and can be considered if monogenetic disease is suspected. Clinicians must be aware that these patients are prone to autoimmune diseases, lymphoproliferative disorders, and malignancies. It must be noted that CVID at present is only treated symptomatically. Management principles involve the use of IRT and judicious use of prophylactic antibiotics.
